# Diagnosing Czech Patients with Inherited Platelet Disorders

**DOI:** 10.3390/ijms232214386

**Published:** 2022-11-19

**Authors:** Jan Louzil, Jana Stikarova, Dana Provaznikova, Ingrid Hrachovinova, Tereza Fenclova, Jan Musil, Martin Radek, Jirina Kaufmanova, Vera Geierova, Eliska Ceznerova, Peter Salaj, Roman Kotlin

**Affiliations:** 1 Centre for Thrombosis and Hemostasis, Institute of Hematology and Blood Transfusion, 128 20 Prague, Czech Republic; 2 Department of Biochemistry, Institute of Hematology and Blood Transfusion, 128 20 Prague, Czech Republic; 3 Laboratory for Disorders in Hemostasis, Institute of Hematology and Blood Transfusion, 128 20 Prague, Czech Republic; 4 Department of Immunomonitoring and Flow Cytometry, Institute of Hematology and Blood Transfusion, 128 20 Prague, Czech Republic; 5 Central Hematology Laboratories, Institute of Medical Biochemistry and Laboratory Diagnostics of the General University Hospital and of the First Faculty of Medicine of Charles University, 128 20 Prague, Czech Republic; 6 Department of Biochemistry and Microbiology, University of Chemistry and Technology Prague, Technicka 5, 166 28 Prague, Czech Republic

**Keywords:** inherited platelet disorders, primary hemostasis, clinical laboratory techniques, *ANKRD26*, *ITGA2B*, NGS

## Abstract

A single-center study was conducted on 120 patients with inherited disorders of primary hemostasis followed at our hematological center. These patients presented a variety of bleeding symptoms; however, they had no definitive diagnosis. Establishing a diagnosis has consequences for the investigation of probands in families and for treatment management; therefore, we aimed to improve the diagnosis rate in these patients by implementing advanced diagnostic methods. According to the accepted international guidelines at the time of study, we investigated platelet morphology, platelet function assay, light-transmission aggregometry, and flow cytometry. Using only these methods, we were unable to make a definitive diagnosis for most of our patients. However, next-generation sequencing (NGS), which was applied in 31 patients, allowed us to establish definitive diagnoses in six cases (variants in *ANKRD26*, *ITGA2B*, and *F8*) and helped us to identify suspected variants (*NBEAL2*, *F2*, *BLOC1S6*, *AP3D1*, *GP1BB*, *ANO6*, *CD36*, and *ITGB3)* and new suspected variants (*GFI1B*, *FGA*, *GP1BA*, and *ITGA2B*) in 11 patients. The role of NGS in patients with suspicious bleeding symptoms is growing and it changes the diagnostic algorithm. The greatest disadvantage of NGS, aside from the cost, is the occurrence of gene variants of uncertain significance.

## 1. Introduction

Inherited platelet disorders (IPDs) are a group of diagnoses involving defects of platelet adhesion or aggregation, insufficient receptor or granule formation and function, and cytoskeletal abnormalities [1]. IPDs not only affect platelets but also occur as a syndromic phenotype and are associated with increased risk of hematological malignancies. IPDs, although rare, account for a significant proportion of inherited bleeding disorders and have a clinical impact on personal health management [2,3,4,5].

The typical clinical symptom in patients with IPDs is bleeding, with severity ranging from mild to life threatening. Bleeding can take the form of unexplained or extensive hematomas, longer nosebleeds that cause anemia or the need for hospital admission, menorrhagia that usually persists from menarche, bleeding from the oral cavity, and high-volume blood loss during delivery, during surgeries, or after tooth extractions [6].

Many IPDs comprise a set of multisystemic disorders, known as syndromic IPDs. Syndromic IPDs are associated with defects in cells, tissues, organs, or the development of neoplastic disease (e.g., Wiskott–Aldrich syndrome, Hermansky–Pudlak syndrome, gray platelet syndrome (GPS), or variant of *ANKRD26*). Patients may present with immunodeficiency, deafness, kidney disease, cataracts, hematological malignancies, skeletal abnormalities, cleft palate, cardiac abnormalities, mental retardation, myopathies, or other abnormalities [1,7]. Despite the availability of guidelines, establishing a definitive diagnosis in some cases remains challenging [8,9,10,11,12].

The first guidelines we used as a template were the 2006 United Kingdom Hemophilia Centre Doctors’ Organization guidelines [8]. These guidelines divided IPDs according to the occurrence of thrombocytopenia, other clinical conditions (e.g., exclude syndromic IPDs), platelet size, and platelet aggregation. To confirm the diagnosis, the use of transmission electron microscopy (TEM), genetic testing, or other specific methods was recommended.

Algorithms and recommendations have changed with the growing role of next-generation sequencing (NGS). Current guidelines (from 2015) recommend NGS as a diagnostic tool [12]. The International Society of Thrombosis and Hemostasis (ISTH) defined gold-standard genes for diagnostics, which are used to interpret the results, as they include most genes suspected of causing IPDs and coagulation deficiencies [12,13]. Up-to-date guidelines are divided into three consecutive steps, from those that are routinely performed to more complex steps, such as TEM or NGS. Small regional or national centers frequently face challenges accessing the recommended methods, as they are often time consuming and expensive.

As first-line diagnostic evaluation of IPDs, the recommended methods are clinical examination, laboratory analysis with general biochemical and coagulation testing, complete blood count, and blood smear, focusing on platelet count, morphology, and function [12]. The first step in clinical examination is obtaining a history of the patient’s bleeding symptoms. Many bleeding scores for the reproducibility of bleeding and standardization have been established. The main score used today is the 2010 ISTH Bleeding Assessment Tool (BAT), which comprises 14 questions on the frequency and severity of symptoms. The questionnaire is administered by a clinician or nurse; however, its objectivity is influenced due to the mostly subjective nature of the answers given by patients [6,14,15,16,17].

Analysis of the functional phenotype of the platelets begins with the use of relatively basic, widespread, and nonspecific tests. Laboratory screening focusing on platelet function should include light-transmission aggregometry (LTA) with standard agonists (ADP, epinephrine, collagen type I, arachidonic acid, and ristocetin) [18], assessment of platelet granule release, and analysis of major platelet surface glycoproteins on resting platelets (GPIIb/IIIa, GP IIIa, GPIb/IX, and GPIb) and on activated platelets (GP IIb/IIIa activation epitope) by flow cytometry [12,19]. Some diagnoses, such as Glanzmann thrombasthenia or Bernard–Soulier syndrome, can be made using these diagnostic tools.

The second step uses LTA with an expanded agonist panel (e.g., thrombin, TRAP6, endoperoxide analogue U46619, collagen-related peptide, CVX, PAR 4 ap, and PAF) and flow cytometry with additional antibodies (GPIa/IIa, GPIV, and GPVI). In addition, measurement of platelet procoagulant activity, necrosis (according to ISTH Platelet Physiology SSC) by incubating samples with high-affinity probes against phosphatidyl serine, and activating cells with calcium ionophore, collagen-related peptide, or combinations of thrombin and collagen may be used. In this step, we can also perform clot retraction, measure serum thromboxane B_2_, and TEM [12].

The third step includes biochemical studies, receptor-binding assays, and molecular genetics studies. Biochemical studies and receptor-binding assays are feasible, but their roles are primarily in verifying NGS findings or confirming specific defects [12].

With the increasing use of NGS in the diagnosis of IPDs and inherited thrombocytopenias, we can obtain a definitive diagnosis. Furthermore, the treatment of IPDs has improved with knowledge of the affected gene or deficiency in platelet function. Recent studies have reported on the use of thrombopoietin receptor agonists. Gene therapy and allogenic stem cell transplantation have been used in some severe forms of IPDs [12].

To this day, many patients in the Czech Republic have a diagnosis of “disorder of primary hemostasis,” which covers different types of IPDs. This imprecise diagnosis affects the preparation of patients for surgery, need for targeted treatment, investigation of family members, and understanding of the pathophysiology of the disease. We decided to investigate patients with suspected IPDs, as we have all the recommended methods available at our center.

The aim of this project is to implement advanced diagnostic methods, such as NGS, in the protocols for IPD diagnostics and contribute to identifying a definitive diagnosis for patients with IPDs.

## 2. Results

### 2.1. Patient Characteristics

**Figure 1:**
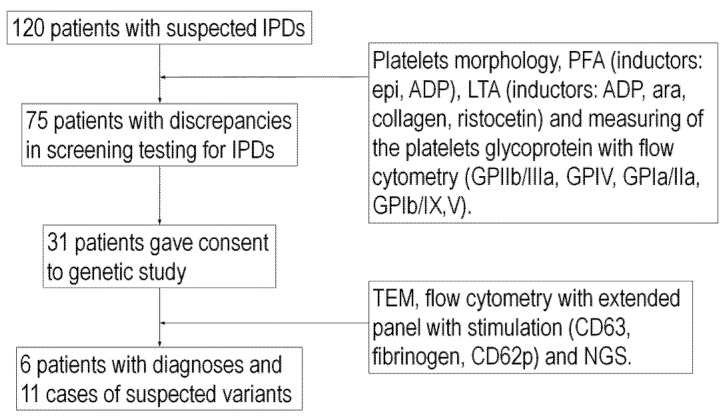
Overview of the study. Abbreviations: ADP—adenosine diphosphate, epi—epinephrine, ara—arachidonic acid, GP—glycoprotein.

First, we identified 120 patients with suspected IPD according to their bleeding symptoms and positive personal or family history. We selected 75 patients who had pathology on routine testing (i.e., morphology, platelet function assay (PFA), LTA, and fluorescence-activated cell sorting (FACS)). Thirty-one patients with suspected IPD (Table 1) gave informed consent to participate in this study and we investigated them using various methods recommended by the ISTH, including NGS (Figure 1).

### 2.2. Bleeding Assessment Tool

We scored 31 patients according to the ISTH BAT (Table 1) [6]. In this group, one child (patient 11) had a pathological BAT score of 5, three patients (all males) had pathological BAT scores, and one male (patient 22) had a normal BAT score. Eighteen (69%) female patients had pathological BAT scores. Nine females had normal BAT scores; we included them in our study because they had pathological results from our screening panel (PFA, LTA, or FACS).

### 2.3. Platelet Morphology

Thrombocytopenia (platelet count <150 × 10^9^/L) was observed in 8 out of 31 (26%) patients. Regarding platelet morphology, large platelets (MPV > 12.8 fL) were observed in seven (23%) patients. In one patient (patient 11), morphology could not be determined because of the small sample volume (Table 1).

### 2.4. Platelet Function Analyzer 

We used the Innovance PFA-200 System to assess platelet function. PFA was normal in eight (26%) patients. Pathological PFA after both inductors (ADP and epinephrine) was observed in 14 (45%) patients. Seven (23%) patients induced by epinephrine and one patient induced by ADP had pathological PFA. One patient (patient 11) was not analyzed because of the small sample volume (Table 2). In all patients, we investigated the level of von Willebrand factor (vWF), which was normal.

### 2.5. Light-Transmission Aggregometry

We used the following inductors for screening investigation: ADP, collagen, ristocetin, and arachidonic acid.

In 16 (51.6%) patients, LTA was slightly decreased after all inductors. In four patients (patient 10, 11, 25, 28), LTA showed decreased aggregation after one inductor (Table 2).

### 2.6. Flow Cytometry

Screening flow cytometry was targeted to platelet GPIIb/IIIa, GPVI, GPIb/IX/V, GPIa/IIa, and P selectin (Table 3). Screening revealed lower expression of at least one glycoprotein in 21 (67.7%) patients. Two patients (patients 4 and 5), who were selected on the basis of clinical examinations, screening results, personal history, and availability of samples, were investigated using flow cytometry with an extended panel of platelet markers in both resting and activated states (Table 3). Five healthy controls were used to verify the extended panel. We employed fluorescence-activated cell sorting (FACS). During analysis, we found that platelets were activated by lysis after stimulation with arachidonic acid and ristocetin in some cases. To avoid this, we decreased the stimulation time from 20 min to 10 min and added 1.5 mM CaCl_2_ to the buffer, which stabilized the platelets.

In patient 4, CD62p marker responses to stimulants were lower in arachidonic acid and slightly lower after ADP. CD63 marker response was higher after EDTA and arachidonic acid and a lower response was observed after ristocetin stimulation. Bound fibrinogen marker response was higher after EDTA stimulation, slightly higher after arachidonic acid and Trap6 stimulation, lower after ristocetin stimulation, and slightly lower after ADP stimulation.

In patient 5, CD62p marker response to stimulants was lower after arachidonic acid and slightly lower after ristocetin stimulation. CD63 marker response was higher after EDTA, slightly higher after arachidonic acid, and lower after ristocetin stimulation. Bound fibrinogen response was higher after EDTA, slightly higher after arachidonic acid and Trap6 stimulation, and slightly lower after ADP stimulation (Table 3).

### 2.7. Whole Mount Transmission Electron Microscopy

**Figure 2:**
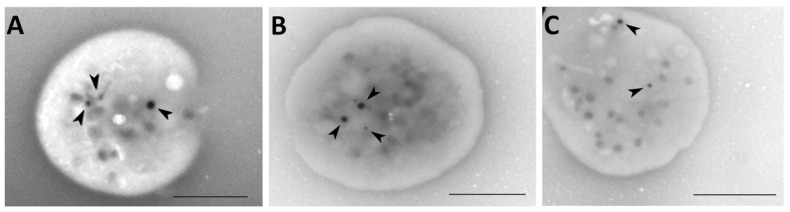
Representative pictures of dense granules (arrows in **A**–**C**) in platelets: (**A**) normal; (**B**) patient 10; (**C**) patient 11. Images were obtained using transmission electron microscopy (TEM; JEM-1010, JEOL, Peabody, MA, USA). Bar scale is 2 µm.

We investigated four patients (patient 4, 5, 10, and 11) using TEM (Figure 2). Patient selection was based on clinical examinations, screening results, and anamnesis. The patients had normal to slightly decreased counts of dense granules (Table 4).

### 2.8. Next-Generation Sequencing

Using NGS, we found a detrimental gene variant (*ANKRD26*, *ITGA2B*, *F8*) in six patients (patients 12, 13, 23, 24, 27, 28; 19%). In one patient (number 26), we found two heterozygous variants associated with Bernard–Soulier syndrome; in 10 patients, we detected different heterozygous variants. NGS results are summarized in Table 5.

## 3. Discussion

Diagnosing IPDs is a complex process and includes not only clinical examination but also morphology, functional testing of platelets, and genetic testing. We followed the current ISTH guidelines from 2015 [12].

As screening methods, we used morphology, PFA, LTA, and FACS and obtained the results described below. These methods did not help us to establish definitive diagnoses that would explain the patients’ symptoms. Therefore, we implemented another method, NGS, to identify causal variants and correlate them with the patients’ phenotypes and diagnoses. 

### 3.1. Morphology 

Morphological examination is a simple and cost-effective way to exclude platelet pathologies, for example, Döhle bodies or other structural abnormalities [21]. We observed thrombocytopenia (platelet count <150 × 10^9^/L) in 8 out of 31 (26%) patients. Regarding platelet morphology, large platelets (MPV >12.8 fL) were observed in seven (23%) patients.

### 3.2. Functional Testing (PFA, LTA, FACS)

PFA is a useful method, especially when the results are repeatedly pathological. It can reveal certain major IPDs or von Willebrand disease (VWD); however, it is necessary to confirm the diagnosis with other methods. In patients for whom the results changed from normal to pathological or vice versa over time, the diagnosis of IPD was not suspected and we instead suspected drug-induced secondary thrombocytopathy. A limitation of this method is a low platelet count; in patients with thrombocytopenia, especially with a platelet count <100 × 10^9^/L, PFA gives false-positive results [22]. Furthermore, normal PFA cannot exclude IPD. For this reason, in patients strongly suspected of having IPD with normal PFA, other investigations were performed. vWF and factor VIII (FVIII) levels were determined in all our patients to exclude VWD. All our investigated patients had normal levels of these factors; only one patient (patient 23) had borderline low levels of FVIII. This patient was identified as a carrier of mild hemophilia A by NGS. Interestingly, her relative (patient 24), who we also identified as carrier of mild hemophilia A, had normal levels of vWF and FVIII.

PFA was pathological after both inductors in 14 (45%) patients (Table 2). Pathological PFA was probably due to thrombocytopenia (<100 × 10^9^/L) in six of the patients (patients 12, 13, 26, 27, 28, and 29). Patient 23 had pathological PFA in contrast with patient 24 (relative of patient 23), who had normal PFA. However, the difference in PFA in these relatives who had the same phenotype might have been drug induced. In six patients (patients 1, 3, 6, 22, 26, and 31), the prolongation of PFA may indicate problems with platelet function. In our cohort, we did not find LTA useful in the diagnostic process, as was indicated in the guidelines [18]. This may be due to the diagnoses we made. We did not obtain a typical picture of each pathology; we mostly observed decreased aggregation after all inductors and the results in each patient changed over time. LTA and NGS were in agreement in only two patients (patients 27 and 28) with a variant of *ITGA2B*.

The advantage of FACS is that it requires only a very small sample. The main disadvantages of FACS are the price of the inductors, especially when using extended panels, and the lack of standardization [23]. Furthermore, our attitude toward this testing changed because of the availability of NGS. We can use FACS to identify not only platelet receptor defects, but also to confirm the clinical impact of identified suspected variants. FACS could be useful in our two cases with the *ANKRD26* variant (patients 12 and 13), as variants in this gene are associated with a typical reduction in GPIb.

### 3.3. Transmission Electron Microscopy

We performed TEM in four patients (patients 4, 5, 10, 11) on the basis of pathological results in first-line tests and bleeding abnormalities. These patients were suspected of having dense granule defects. We observed slightly lower counts of dense granules compared with normal counts (Table 4) [20]. However, TEM is not suitable for routine laboratory use because it is time consuming and expensive. In addition, centers that do not have TEM equipment require cooperation from research facilities or departments.

### 3.4. Next-Generation Sequencing

**Figure 3:**
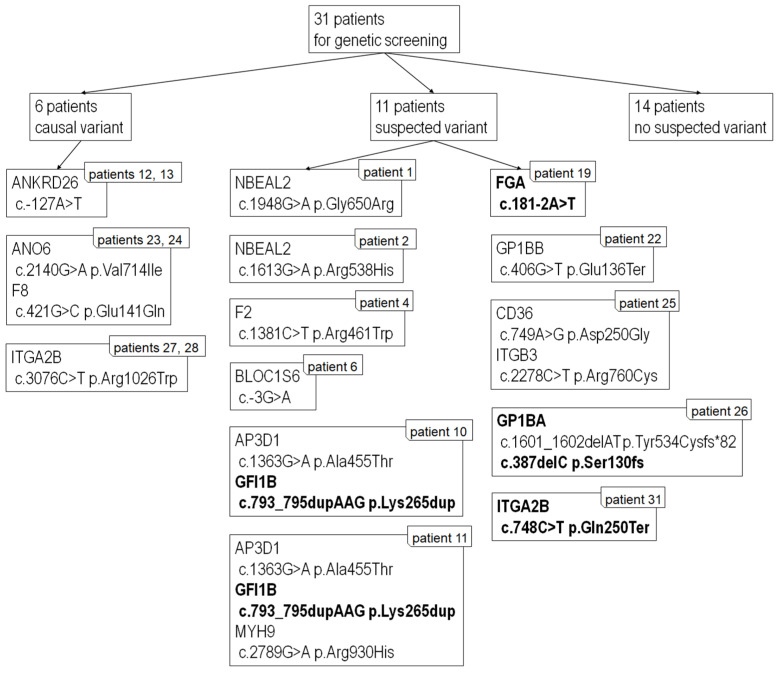
Summary of NGS results. Novel variants are in bold. All variants are heterozygous. For reference sequences and other information see Table 5.

We used NGS to investigate 31 patients with bleeding symptoms with a suspected diagnosis of IPD. We found clinically significant variants in 6 patients (patients 12, 13, 23, 24, 27, and 28) and we found highly suspicious variants in 11 other patients; however, 14 patients remained without a detected causal variant to explain their bleeding problems (Table 5, Figure 3). In the description, we focused mainly on the six patients in whom the identified variants fully explained their phenotype.

Due to the number of genes screened and the amount of data obtained by NGS, we do not report polymorphisms found in our patients. Detected polymorphisms were not clinically relevant and their benign status was confirmed primarily by their high prevalence in population.

Through genetic testing, we identified a heterozygous variant of the *ANKRD26* gene (c.-127A>T) in two related patients (patients 12 and 13). Variants of *ANKRD26* and of transcription factors *ETV6* and *RUNX1*, which are associated with autosomal dominant (AD) inherited disorders, represent almost one-quarter of inherited thrombocytopenia cases [24,25,26]. According to the World Health Organization (WHO) 2016 revision, these variants are classified as myeloid neoplasms with germline predisposition and pre-existing platelet disorders [27,28]. Patients with *ANKRD26* variants have mostly variable bleeding symptoms, moderate-to-severe thrombocytopenia with normal-sized platelets, and reductions in α-granules and platelet GPIb. Platelet aggregation is normal in these patients and they have elevated serum thrombopoietin. Variants detected in the *ANKRD26* gene are one of the most prevalent causes of inherited thrombocytopenia; however, only a small number of these patients has been identified worldwide [24,25,26].

These findings correlated well with results from patients 12 and 13. Both patients had positive BAT scores; however, there was a difference in their phenotypes, where patient 13 had a lower score than patient 12 (Table 1). Both had thrombocytopenia, but patient 12 had slightly larger platelets and suffered from more severe chronic anemia due to heavier menstrual bleeding. Both patients had pathological PFA after both inductors, which we credited to thrombocytopenia. Both patients had identical results following FACS (low GPIIb/IIIa, GPIa/IIa, and GPVI; normal GPIb/IX/V) (Table 2).

The father of these two patients was probably also a carrier of the detected *ANKRD26* variant. He was treated incorrectly for immune thrombocytopenic purpura, for which he underwent splenectomy and subsequently died of surgery- and immunosuppression-related sepsis. This case confirms the need to establish a correct diagnosis and the importance of investigating family members.

We will investigate the relatives of patients 12 and 13 in the future and the patients will be routinely assessed in our ambulance due to a higher risk of progression to acute myeloid leukemia (AML).

Other patients with confirmed diagnosis of IPD were two related patients with an identified heterozygous variant of *ITGA2B* c.3076C>T (p.Arg1026Trp) (patients 27 and 28) (Table 5). This variant sufficiently explained the phenotype of these patients and was already known. It causes AD thrombocytopenia with normal platelet size [29]. The same variant had been previously reported in seven Japanese families with AD thrombocytopenia; however, all of these patients had macrothrombocytopenia [30,31]. There has been a report of a four-generation family in the USA with 10 individuals with AD thrombocytopenia and normal platelet size [29]. Our two patients also had thrombocytopenia with normal platelet volume.

The phenotype of these two patients was similar in most parameters. The only difference was that patient 28 had episodes of severe thrombocytopenia in his medical history, which were treated with corticosteroids. During observation of this patient in our center, the treatment was gradually discontinued without any impact on the patient’s platelet count. Bleeding scores in these two patients were slightly elevated (BAT score 4) (Table 1). 

Patients 27 and 28 had pathological PFA after both inductors, which may have been due to thrombocytopenia. There was a similar observation in LTA, where both patients had lower response to arachidonic acid and ADP. This finding is in line with other studies [32]. Furthermore, the patients had low levels of GPIIb/IIIa and GPVI on FACS. 

Surprisingly, we identified two related patients (patient 23 and 24) with a variant associated with a mild form of hemophilia A (HA, c.421G>C p.Glu141Gln). We primarily examined these patients because of suspected IPD as both had bleeding symptoms. Only one of the patients (patient 23) had borderline FVIII; otherwise, their levels of FVIII and vWF were normal. One of the patients delivered a boy with mild HA during our study. The pediatricians were informed of the potential risks and no complications occurred during or after delivery. The BAT bleeding scores were slightly elevated: 8 in patient 23 and 6 in patient 24. Both had normal platelet count and size (Table 1). There was a difference in PFA (pathological in patient 23, normal in patient 24); in LTA, there was a slightly reduced answer to all inductors in patient 23 and borderline in patient 24. Low expression of GPVI and GPIIb/IIIa was observed in patient 23 and patient 24 had low expression of GPIb/IX/V (Table 2). In concordance with the diagnosis of suspected IPD, we also found a heterozygous variant in the gene *ANO6* in these patients (Table 5). These two cases demonstrate that, during investigation of patients for IPD, we can also detect other diagnoses causing bleeding problems. Therefore, it is important to examine patients who have bleeding abnormalities and additional inherited diagnoses to exclude syndromic IPD.

We identified two unrelated patients with mild-to-moderate forms of Noonan syndrome (patients 1 and 6). This syndrome is a genetic disorder that prevents normal development in various parts of the body and is associated with a variety of symptoms, such as unusual facial characteristics, short stature, heart defects, other physical problems, and possible developmental delays. Management of Noonan syndrome focuses on controlling symptoms and complications, and growth hormones may be used to treat short stature in some patients. Coagulation problems, such as abnormal susceptibility to bleeding, often referred to as bleeding diathesis, were described in these patients. Bleeding diathesis may be related to vascular, platelet, or coagulation defects [33].

The two patients with predominant heart defects had moderate bleeding abnormalities according to their BAT scores (patient 1 had BAT score 4 and patient 6 had BAT score 8), which was why they were referred for examination. They had normal platelet count, but patient 1 had larger platelets (Table 1). They had pathological PFA after both inductors, which may have indicated IPD, as they both had normal platelet counts. Patient 6 had slightly decreased LTA after all inductors and their other results were normal. Using NGS, we found different variants in these patients, which may explain the different phenotypes. In patient 1, a heterozygous variant in *NBEAL2* was discovered; in patient 6, a heterozygous variant in *BLOC1S* was identified (Table 5). Variants of the *BLOC1S* gene are associated with Hermansky–Pudlak syndrome type 9.

These data indicate that it is worth investigating patients with bleeding problems and other symptoms, especially as a part of congenital syndromes.

We identified two different heterozygous variants of the *NBEAL2* gene in two patients (patient 1 and patient 2). Variants in this gene cause GPS, an autosomal recessive disorder characterized by a deficit of α-granules (number and/or content). Patients usually present with moderate thrombocytopenia, with large, pale platelets and moderate mucocutaneous hemorrhagic diathesis. GPS is a progressive syndrome, usually developing into severe thrombocytopenia during adolescence and adulthood, with a gradual degree of myelofibrosis and rarely splenomegaly. Some patients have leukopenia or are prone to autoimmune diseases, have defective NETosis, and develop autoantibodies [34,35,36]. Patient 1 was described in the section about Noonan syndrome. Patient 2 had a high BAT score of 14 and their platelet number and size were normal (Table 1); PFA was normal, LTA was slightly low after all inductors, and there was decreased expression of GPIIb/IIIa (Table 2).

Patient 26 was a combined heterozygote of two variants of the *GP1BA* gene (p.Tyr534Cysfs and p.Ser130fs). She had macrothrombocytopenia and bleeding symptoms (BAT score 8) (Table 1). She had pathological PFA after both inductors, low response after all inductors (especially ristocetin) in LTA, and low GPIb/IX/V in FACS, which correlates with a potential diagnosis of Bernard–Soulier syndrome (Table 2).

In eight patients (patients 4, 10, 11, 19, 22, 25, 26, and 31), we found different variants of platelet genes and coagulation factor genes that did not sufficiently explain the patients’ phenotypes and bleeding symptoms and require further investigation (Table 5).

Limitations of our study include the availability of the recommended methods and their costs. We used TEM, FACS, and NGS as they were available to us. We observed possible false positivity in FACS in GPVI (Table 2), which was probably caused by a lack of standardization of this method. We did not assess platelet granule release because we have not established this method in our institution. NGS gave us better results in terms of identification of gene variants and linked syndromes. NGS enabled a molecular diagnosis in 55% (17/31) of our patients. Comparing our results with the available literature, a Spanish group identified a causal variant in 70% of their patients [37] and German authors were successful in 40% of their cases [38]. We found 19 different candidate variants in 14 genes and 4 variants in genes *GFI1B*, *GP1BA*, *FGA*, and *ITGA2B* were not previously described (Table 5). We found an additional four variants in genes *BLOC1S6*, *GP1BB*, *ANO6*, and *CD36*, which were mentioned only in the NCBI database, where only information about incidence in population was provided, with no further information about genotype. Our results were comparable to those of the Spanish and German groups. We could make a diagnosis that clearly correlated with clinical phenotype in 19% of our patients; in another 35% of patients, we found suspected variants, which require further investigation.

For patients in whom we did not find a causal/suspected variant, we will continue with our investigation, including NGS using different panels of genes. According to our study, the position of NGS is advancing in the diagnostic process. From our experience, we support its place in the second step in patients presenting with a phenotype strongly indicative of IPDs. We find NGS highly sensitive compared with all available methods and the only disadvantage is the occurrence of variants of unknown significance. The conventional methods will retain their importance; however, we believe that NGS will be used more often to confirm borderline results, or we will be able to confirm diagnoses made by NGS using international registries.

## 4. Materials and Methods

### 4.1. Patient Group

Between 2015 and 2021, we selected a group of 120 patients with suspected IPD according to their bleeding symptoms. From this cohort, 75 patients had some discrepancies in screening testing for IPDs. These tests included morphology (number and size of platelets), PFAs (inductors: epinephrine and ADP), LTA (inductors: ADP, arachidonic acid, collagen, and ristocetin), and measuring of the platelet’s glycoproteins with flow cytometry (GPIIb/IIIa, GPVI, GPIa/IIa, GPIb/IX/V, and P selectin). 

Of these 75 patients, 31 patients (Figure 1) consented to genetic study. All the samples were obtained and analyzed in accordance with the Ethical Committee regulations of the Institute of Hematology and Blood Transfusion, Prague, Czech Republic (reference number: 12 February 2016). Prior to enrollment in the study, informed consent was obtained from each subject. All data were analyzed anonymously. The study was carried out in accordance with International Guidelines and the Declaration of Helsinki.

### 4.2. Materials

Human genomic DNA was isolated from leukocytes from peripheral whole blood [39].

All the reagents employed were of analytical grade or higher purity and their source and specification are described.

### 4.3. Bleeding Assessment Tool

We scored all of our 31 patients according to ISTH-BAT score, with a defined cut-off for a positive or abnormal BAT score of ≥4 in adult males, ≥6 in adult females, and ≥3 in children [16]. 

### 4.4. Platelet Function Assay 

We used the Platelet Function Analyzer (PFA-200, Siemens Healthineers, Oxford, UK) to evaluate platelet adhesion and aggregation induced simultaneously by a high flow rate (4000–6000 s^−1^) and by a mixture of collagen with ADP or with epinephrine in the membrane. The inductors were also obtained from Siemens Healthineers, Oxford, UK. The test is highly sensitive for the screening of severe IPD or VWD [22].

### 4.5. Light-Transmission Aggregometry

LTA is considered the gold standard for platelet function testing [18]. We used a PAP-8 aggregometer (BioData Corp., Horsham, PA, USA), eight-channel optical aggregometer, and inductors ADP and ristocetin (Helena Laboratories, Beaumont, TX, USA), arachidonic acid, and collagen (Hyphen BioMed, Neuville-sur-Oise, France). Panel of inductors: 20 μmol/l ADP, 1.25 μg/mL collagen, 1.2 mg/mL ristocetin, and 1.0 mmol/l arachidonic acid (all final concentrations in PRP).

### 4.6. Flow Cytometry

Screening flow cytometry was targeted to platelet glycoproteins GPIIb/IIIa, GPVI, GPIb/IX/V, GPIa/IIa, and P selectin (Table 2). We performed our screening tests using a Navios flow cytometer (Beckman Coulter, Brea, CA, USA) and the data were analyzed using the program Kaluza (Beckman Coulter, Brea, CA, USA). The cytometer was set by EuroFlow standard operational process (SOP) for setting our diagnostic tools and compensations, according to Kalina et al. [40].

We used antibodies for detection of platelet glycoproteins: anti-CD62P, clone LYP20 (Stago, category number: 01022), anti-GPVI, clone 1G5 (Stago, cat. nr.: 01083), anti-CD41, clone P2 (Beckman Coulter, Brea, CA, USA, cat. nr.: IM0145), anti-CD42a, clone SZ1 (Beckman Coulter, Brea, CA, USA, cat. Nr.: IM0538), and anti-CD49b, clone Gi9 (Beckman Coulter, Brea, CA, USA, cat. Nr.: IM0717).

We used a Platelet Calibrator calibration kit (Stago, Asnières-sur-Seine, France, cat.nr.: 00457), which included negative isotype controls IgG1 and IgG2a, calibration beads with known number of ABC, and secondary antibodies anti-IgG FITC for their quantification.

We set our normal values for GPIIb/IIIa 60,000 ± 12,000, GPVI 7700 ± 800, GPIb/IX/V 30,500 ± 4000, GPIa/IIa 4500 ± 1500, and P selectin under 1000 sABC. 

FACS was used for detection of surface glycoproteins and functional testing of platelets in extended panel. We used a BD LSR Fortessa flow cytometer (BD Biosciences, San Diego, CA, USA) and inductors TRAP-6 (Abcam Plc., Shanghai, China), ADP, ristocetin (Helena Laboratories, Beaumont, TX, USA), and arachidonic acid (Hyphen-Biomed, Neuville-sur-Oise, France). Software for analyzing flow cytometry was FlowJo 10.6.1. (FlowJo LLC, Ashland, OR, USA).

For platelet stimulation, we used the amount of inductors according to work of Rubak et al. [19]. We looked for signs of activation according to the presence of markers CD63 and CD62p.

### 4.7. Whole-Mount Transmission Electron Microscopy

Whole-mount samples of platelets were prepared within 3 h of blood collection. Whole blood from healthy donors or patients was collected in acid citrate dextrose (ACD) tubes and stored at room temperature. Platelet-rich plasma (PRP) was separated from whole blood by centrifugation at 200× *g* for 15 min at 37 °C. Small drops of PRP were placed on 300-mesh, formvar-coated grids, rinsed within 10–15 s with drops of distilled water, and dried from the edge with pieces of filter paper. Four grids were prepared for each patient. The grids were inserted into a TEM (JEOL JEM-1010, Peabody, MA, USA). Dense granules from platelets were counted based on interpretation criteria published by Chen et al. [41]. Normal counts were between 3 and 8 dense granules per platelet [20]. Dense granules were counted in 200 platelets in each patient.

### 4.8. Next-Generation Sequencing Analysis and Variant Classification

A customized gene panel was designed to target all exons and intronic flanking regions of 42 genes involved in platelet disorders and as recommended by ISTH in TIER1 (*vWF*, *MYH9*, *GP1BA*, *GP1BB*, *GP9*, *WAS*, *MPL*, *ITGA2B*, *ITGB3*, *HPS1*, *HPS3*, *HPS4*, *HPS5*, *HPS6*, *AP3B1*, *AP3D1*, *BLOC1S3*, *BLOC1S6*, *DTNBP1*, *LYST*, *ANKRD26*, *NBEAL2*, *GFI1B*, *CD36*, *GP6*, *ANO6*, *P2RY12*, *TBXA2R*, *MLPH*, *MYO5A*, *PLAU*, *RAB27A*, *VPS33B*, *VIPAS39*, *PLA2G4A*, *RGS2*, *ADRA2A*, *ADRA2B*, *F2R*, *F2RL3*, *ITGA2*, and *ITGB1*). Our panel was designed using Agilent SureDesign software using GRCh38 as the reference (version 7.8.2.9, Santa Clara, CA, USA).

For library preparation, we used 130 ng of genomic DNA from each sample. Amplification and library construction were performed using an SSEL XT Low Input Reagent Kit (Agilent Technologies, Santa Clara, CA, USA). All indexed amplicons were pooled in a single tube and underwent adapter ligation and clean up. The NGS libraries were simultaneously sequenced using a MiSeq Reagent Kit v3 (150 cycles) on a MiSeq system (Illumina, San Diego, CA, USA).

The NGS pipeline output, de-multiplexed, paired sequence files in fastq format were used as input for analysis. After variant calling, the resulting vcf files were used as an input to annotate all variants. Then, we used the following criteria to identify the candidate disease-causing variants and to filter out polymorphisms: frequency <1% for the European population based on minor allele frequency (MAF), international databases of disease-causing variants for each gene (if available), and variants’ influence on coding areas. In accordance with the American College of Medical Genetics and Genomics and the Association for Molecular Pathology (ACMG/AMP) guidelines, the potential pathogenic effect of identified variants was assessed using VarSome software. We also used other in silico tools to determine whether the identified variants were pathogenic or benign: PMut, MutPred2, PolyPhen2, PANTHER, Mutation Taster, Provean, SIFT, and SNPs&GO. We assessed the variant as potentially causal if at least six out of the eight software packages described the variant as pathogenic.

### 4.9. Statistical Analysis

Data were processed and the mean was calculated using MS Office Excel (MS Office 2016, Microsoft Corp., Redmond, WA, USA).

## 5. Conclusions

Despite being a time- and resource-consuming process, NGS could be a decisive tool in providing a definitive diagnosis in patients with IPDs if correctly applied, i.e., used in patients with a strongly indicative phenotype or with pathological results from conventional methods. We confirmed the efficacy of NGS as a tool in diagnosing patients with IPD in differential diagnoses. NGS helped us to confirm a definitive diagnosis in 19% of our patients and to refine the diagnosis in 35%. Our results indicate the potential of NGS in making correct diagnoses. Despite the disadvantages of NGS, namely the occurrence of variants of unknown significance, we believe that its role will become more prominent. As IPDs are rare diseases, it is important to centralize the national and international data and form multidisciplinary teams in national coagulation centers.

## Figures and Tables

**Table 1:** Patients’ Bleeding Assessment Tool (BAT) scores and platelet morphological features.

Patient No.	Age	Sex	BAT Score	Thrombocytopenia	Size of Platelet
1	34	F	4	No	Macro
2	53	F	14	No	Normal
3	63	F	9	No	Normal
4	31	F	6	No	Normal
5	36	F	9	No	Normal
6	32	F	8	No	Normal
7	37	M	8	Yes	Macro
8	45	F	8	No	Macro
9	28	F	6	No	Normal
10 ^a^	42	F	11	No	Macro
11 ^a^	8	M	5	NA	NA
12 ^b^	44	F	11	Yes	Macro
13 ^b^	49	F	6	Yes	Normal
14 ^c^	48	F	10	No	Normal
15 ^c^	25	F	1	No	Normal
16	48	F	5	No	Normal
17	29	F	7	No	Normal
18	32	F	10	No	Normal
19	59	F	4	No	Normal
20	20	F	21	No	Normal
21	62	F	3	No	Normal
22	41	M	2	Yes	Macro
23 ^d^	53	F	8	No	Normal
24 ^d^	25	F	6	No	Normal
25	66	F	5	No	Macro
26	27	F	8	Yes	Macro
27 ^e^	61	M	4	Yes	Normal
28 ^e^	40	M	4	Yes	Normal
29	30	F	5	Yes	Macro
30	28	F	4	No	Normal
31	35	F	8	No	Normal

Abbreviations: NA—not available, F—female, M—male, Macro—macro thrombocytes; ^a–e^—indicate members of the same family. Pathological BAT: adult males ≥ 4, adult females ≥ 6, children ≥ 3. Normal platelet size (MPV): 7.8–12.8 fL.

**Table 2:** Results of platelet function analysis.

Patient No.	PFA	LTA (ADP, Arachidonic Acid, Collagen, Ristocetin)	Flow Cytometry
1	pathological (epi, ADP)	Normal	Normal
2	Normal	low nonspecific	low GPIIb/IIIa
3	pathological (epi)	low nonspecific	low GPIIb/IIIa, GPIb/IX/V a GPVI
4	Normal	Normal	low GPVI
5	pathological (epi)	low nonspecific	NA
6	pathological (epi, ADP)	low nonspecific	Normal
7	pathological (epi)	Normal	low GPVI, GPIb/IX/V, GPIa/IIa
8	pathological (epi, ADP)	slightly low	low GPIIb/IIIa
9	pathological (epi)	Normal	slightly low GPVI
10 ^a^	Normal	slightly low ADP	low GPVI
11 ^a^	NA	low ADP, ara	NA
12 ^b^	pathological (epi, ADP)	NA	low GPIIb/IIIa, GPIa/IIa, GPVI
13 ^b^	pathological (epi, ADP)	NA	low GPIIb/IIIa, GPIa/IIa, GPVI
14 ^c^	pathological (epi)	low nonspecific	Normal
15 ^c^	pathological (epi, ADP)	slightly low nonspecific	Normal
16	pathological (epi, ADP)	low nonspecific	low GPVI, GPIIb/IIIa
17	pathological (epi, ADP)	Normal	NA
18	Normal	slightly low nonspecific	Normal
19	Normal	Normal	low GPVI, GPIIb/ IIIa
20	Normal	slightly low nonspecific	NA
21	pathological (epi)	slightly low	low GPIIb/IIIa and GPVI, slightly low GPIa/IIa
22	pathological (ADP)	Normal	Normal
23 ^d^	pathological (epi, ADP)	slightly low nonspecific	low GPVI, GPIIb/IIIa
24 ^d^	Normal	Borderline	low GPVI, GPIb/IX/V, GPllb/IIIa
25	Normal	low mainly ADP, ara	low GPVI, GPIa/IIa
26	pathological (epi, ADP)	low, mainly ristocetine	low GPIb/IX/V
27 ^e^	pathological (epi, ADP)	very low ara, slightly low ADP	low GPIIb/IIIa, GPVI
28 ^e^	pathological (epi, ADP)	slightly low ADP, ara	low GPIIb/IIIa a GPVI
29	pathological (epi, ADP)	NA	low GPVI
30	pathological (epi)	Normal	slightly low GPVI
31	pathological (epi, ADP)	slightly low, mainly ADP	low GPIIb/IIIa, GPVI

Abbreviations: ADP—adenosine diphosphate, epi—epinephrine, ara—arachidonic acid, GP—glycoprotein, NA—not available; ^a–e^—indicate members of the same family. LTA: normal—>60%, slightly low—60–40%, low—40–10%, very low—<10%.

**Table 3:** Results of flow cytometry with an extended panel of inductors.

	Patient 4			Patient 5			Controls (n = 5)		
Stimulant/Marker	CD62p	CD63	Bound Fbg	CD62p	CD63	Bound Fbg	CD62p *	CD63 *	Bound Fbg *
EDTA	1.30	2.55	3.37	2.16	8.77	3.68	1.11	0.99	0.02
							(0.46–1.75)	(0.92–1.06)	(0–0.04)
ADP	55.60	32.40	31.50	51.40	38.40	32.50	66.64	26.74	45.66
							(56.47–78.81)	(21.24–32.24)	(37.72–53.6)
Ara	7.79	57.60	60.00	21.20	55.70	57.20	60.04	26.10	32.26
							(45.5–74.58)	(13.86–38.34)	(11.27–53.25)
Ristocetin	93.30	8.84	98.90	88.40	13.70	98.90	99.66	39.82	99.76
							(99.47–99.85)	(23.86–55.78)	(99.59–99.93)
Trap-6	90.30	80.80	66.20	46.20	45.50	15.50	87.82	77.96	40.34
							(82.15–93.49)	(67.19–88.73)	(30.19–50.49)

* Values in brackets represent the 95% confidence interval. Abbreviations: Fbg—fibrinogen, Ara—arachidonic acid, ADP—adenosine diphosphate, EDTA—ethylenediaminetetraacetic acid, Trap6—thrombin receptor activator peptide.

**Table 4:** Results of TEM.

Patient No.	Number of Dense Granules *
4	2.00 ± 1.91
5	2.39 ± 1.95
10	2.89 ± 2.24
11	2.08 ± 1.49

* The values are presented as mean ± SD. Normal range is 3–8 granules per platelet [20].

**Table 5:** NGS results of patients.

Patient No.	Gene	Variant		Reference Sequence	Disease Associated with the Gene	NCBI
1	NBEAL2	het.c.1948G>A	p.Gly650Arg	NM_015175.3	Gray platelet sy	rs201373710
2	NBEAL2	het.c.1613G>A	p.Arg538His	NM_015175.3	Gray platelet sy	rs368310677
4						
	F2	het.c.1381C>T	p.Arg461Trp	NM_000506.5	FII deficiency	rs121918478
6	BLOC1S6	het.c.-3G>A	-	NM_012388.4	Hermansky-Pudlak sy type 9	rs746824320 ^¥^
10 ^a^	AP3D1	het.c.1363G>A	p.Ala455Thr	NM_004188.8	Hermansky-Pudlak sy	rs200459002
	GFI1B	**het.c.793_795dupAAG**	**p.Lys265dup**	NM_004188.8	Gray platelet sy	---
11 ^a^	AP3D1	het.c.1363G>A	p.Ala455Thr	NM_003938.8	Hermansky-Pudlak sy	rs200459002
	GFI1B	**het.c.793_795dupAAG**	**p.Lys265dup**	NM_004188.8	Gray platelet sy	---
	MYH9	het.c.2789G>A	p.Arg930His		MYH9RD	rs727504740
12 ^b^	ANKRD26	het.c.-127A>T	-	NM_014915.3	-	---
13 ^b^	ANKRD26	het.c.-127A>T	-	NM_014915.3	-	---
19	FGA	**het.c.181-2A>T**	-	NM_000508.5	dysfibrinogenemia	---
22	GP1BB	het.c.406G>T	p.Glu136Ter	NM_000407.5	Bernard-Soulier sy	rs953345181 ^¥^
23 ^d^	ANO6	het.c.2140G>A	p.Val714Ile	NM_001025356.3	Scott sy	rs765525912 ^¥^
	F8	het.c.421G>C	p.Glu141Gln	NM_000132.3	Hemophilia A	---
24 ^d^	ANO6	het.c.2140G>A	p.Val714Ile	NM_001025356.3	Scott sy	rs765525912 ^¥^
	F8	het.c.421G>C	p.Glu141Gln	NM_000132.3	Hemophilia A	---
25	CD36	het.c.749A>G	p.Asp250Gly	NM_000072.3	GPIV deficiency	rs1375879584 ^¥^
	ITGB3	het.c.2278C>T	p.Arg760Cys	NM_000212.3	Glanzmann thrombasthenia	---
26	GP1BA	het.c.1601_1602delAT	p.Tyr534Cysfs*82	NM_000173.7	Bernard-Soulier sy	rs763978422
	GP1BA	**het.c.387delC**	**p.Ser130fs**	NM_000173.7	Bernard-Soulier sy	---
27 ^e^	ITGA2B	het.c.3076C>T	p.Arg1026Trp	NM_000419.5	Glanzmann thrombasthenia	rs147265794
28 ^e^	ITGA2B	het.c.3076C>T	p.Arg1026Trp	NM_000419.5	Glanzmann thrombasthenia	rs147265794
31	ITGA2B	**het.c.748C>T**	**p.Gln250Ter**	NM_000419.5	Glanzmann thrombasthenia	---
3, 5, 7, 8, 9, 14 ^c^, 15 ^c^, 16, 17, 18, 20, 21, 29, 30-no suspected causal variant detected						

Abbreviations: het—heterozygous, sy—syndrome, MYH9RD—MYH9-related disease; ^a–e^—indicate members of the same family. Novel variants (not found in databases LOVD^3^, ClinVar, NCBI) are in bold, ^¥^—variants found only in NCBI with no further information about genotype. Polymorphisms are not listed.

## Data Availability

The data presented in this study are available on request from the corresponding author.
